# Production of surfactin from *Bacillus subtilis *MZ-7 grown on pharmamedia commercial medium

**DOI:** 10.1186/1475-2859-6-17

**Published:** 2007-06-05

**Authors:** Muaaz Mutaz Al-Ajlani, Muhammad Abid Sheikh, Zeeshan Ahmad, Shahida Hasnain

**Affiliations:** 1 Department of Microbiology and Molecular Genetics, University of the Punjab, Quaid-e- Azam Campus. Lahore, Pakistan

## Abstract

**Background:**

Commercial medium (Pharmamedia) was investigated for the production of surfactin by *Bacillus subtilis *MZ-7. Different media (defined, semi-defined, and complex media) were compared for the production of surfactin after fixing the least influential variables in standardized fermentation conditions. Carbohydrate and nitrogen supplements were also tried to improve production in Pharmamedia.

**Results:**

Surfactin production was confirmed using PCR along with other analytical techniques and monitored by RP-HPLC and MALDI-TOF-MS. We found that optimized and brain heart infusion media were best for production of surfactin (280 mg/L) and a relatively comparable production with Pharmamedia (220 mg/L), however, supplementing Pharmamedia with Fe^+ ^(4.0 mM) and sucrose (2 g/L) leads to a maximum production of about (300 mg/L).

**Conclusion:**

Cottonseed-derived medium proved to be a suitable substrate for the production of bioactive substances including surfactin, a useful compound in both medical and biotechnological fields. The medium provided not only higher product accumulations but at considerably lower cost with potential for large scale industrial applications.

## 1. Background

*Bacillus subtilis *strains produce a broad spectrum of bioactive peptides with great potential for biotechnological and biopharmaceutical applications. A well-known class of such compounds includes the lipopeptides surfactin [1-3], fengycin [4], and the iturin compounds [5] iturin A, B, and C [6], mycosubtilins [7], and bacillomycins [8], which are amphiphilic membrane-active biosurfactants and peptide antibiotics with potent antimicrobial activities. Although surfactin was discovered four decades ago, there has been a revival of interest, triggered by increasing evidence for their medical effectiveness [9,10]. Surfactin possessed antimicrobial [11-13], antiviral [14-16], antitumor [17], hemolytic [18], blood anticoagulant, and fibrinolytic [19,18] activities. As one of the strongest biosurfactants [20,21], surfactin has numerous environmental and biotechnological applications [22] and has shown particular utility in oil recovery [23,24], remediation of soil contaminated by heavy metals [25,26], and biocontrol against phytopathogens [27,28] and insects [29]. Diverse new properties have been recognized, including emulsification, foaming [30], inhibition of starfish oocyte maturation [31], and antimycoplasmic activities [32].

Despite many advantages of surfactins over chemical agents and great recent advances in our understanding of surfactins [33-37], there have been hardly any significant applications of surfactins, mainly because of low strain productivity and the need for expensive substrates [38,39]. Efforts must therefore be redirected to improve production efficiency and recovery bioprocesses in order to optimize yields [38]. An earlier study carried out using nutrient broth gave a very low yield (100 mg/L) [3]. A minimal mineral salts medium was later defined, containing NH_4_NO_3 _(0.05 M) as the inorganic nitrogen source and glucose (4%) as the carbon source (Cooper's medium; [40]). Other studies have used a semisynthetic medium (Landy's medium; [41]) that contains L-glutamic acid (5 g/L) as the organic nitrogen source, glucose (2%) as the carbon source, and trace metals [42,43], in one case supplemented with 0.1% yeast extract and 2 mg/l phenylalanine [15]. Optimizing the mineral requirement was particularly beneficiary for the production of surfactin [44,45,40]. Finally, potential substrates for surfactant production have been sought that might provide cheaper and renewable sources for economical production [38,46]. Interesting cheap sources have been described from agroindustrial crops and residues, such as cassava [47], soybean, sugar beet, potato [48,49], and sweet sorghum, from crop residues such as bran and straw of wheat and rice, and from waste derived from oil processing mills, for example soy molasses from soybean processing and the crude glycerol fraction from biodiesel production [50]. There have been no detailed reports concerning other potentially economical substrates, most notably renewable resources, such as vegetable oils, distillery and dairy wastes [51].

In this report, we describe surfactin production by two producer strains and one non-producer strain of *Bacillus subtilis *in synthetic medium and cottonseed-derived medium (Pharmamedia) and demonstrate the potential of the latter to give similar yields as more expensive media.

## 2. Materials and methods

### 2.1 Microorganisms

*B. subtilis *MZ-7 [52] is an environmental isolate (the sequence of its 16S rRNA is found in accession no. DQ327713). *B. subtilis *ATCC 21332 [3] and *B. subtilis *subsp. *subtilis *168 [53] are surfactin and non-surfactin producers, respectively [54]. The later strain acted as a negative control in all experiments. *Bacillus fusiformis *NRS 613^T ^was regularly used as a test organism. All strains except strain MZ-7 were obtained from the Bacillus Genetic Stock Center, Ohio, USA.

### 2.2. Media and culture condition

Pharmamedia (Traders Protein, USA) is an economical, finely ground, yellow flour made from the embryo of cottonseed. Its detail composition as provided by the manufacturer is listed in Table 1. Landy medium [41], Cooper's medium [40], optimized medium [55], modified Landy medium [55], and minimal salt medium [56] were made as previously described. Sugars (2 g/L) and nitrogen supplements (3 g/L) were made to the control medium. In all cases, pre-inoculum was prepared by suspending in 10 ml deionized water colonies taken from minimal salt agar. The suspensions were adjusted to 5 × 10^12^cells per ml. Growth temperature and pH values were maintained at 35°C and 7 ± 0.5, respectively. Fermentation was carried out for 24 or 48 hours in 250 ml conical flasks containing 50 ± 5 mL broth on an orbital shaker operating at 120 rev/min.

**Table 1:** Chemical characteristics of Pharmamedia

**ANALYSIS (TYPICAL)**			
Total Solids		98.18%	
Protein (N × 6.25)		58.76%	
NFE (Carbohydrates)		24.13%	
Reducing Sugars		1.18%	
Nonreducing Sugars		1.16%	
Fat (Linoleic, Oleic and			
Palmitic Fatty Acids		4.25%	
Ash		6.71%	
Fiber		2.55%	
Moisture		1.82%	
Gossypol (A Yellow Pigment)		0.04%	
pH (Aqueous Solution)		6.5	

**AMINO ACIDS**			

Lysine	4.44%	Alanine	4.10%
Histidine	2.93%	1/2 Cystine	1.67%
Arginine	12.35%	Valine	4.54%
Aspartic Acid	9.69%	Methionine	1.57%
Threonine	3.47%	Isoleusine	3.31%
Serine	4.77%	Leucine	6.34%
Glutamic Acid	22.41%	Tyrosine	2.87%
Proline	4.25%	Phenylalanine	5.71%
Glycine	4.41%	Tryptophan	1.17%

**SOLUBLES**			

Total Solubles		37.20%	
Soluble Amino Nitrogen		0.023%	
Soluble Phosphorus (P)		0.702%	
Soluble Iron (Fe)		ND	
Soluble Magnesium (Mg)		0.391%	

**MINERAL CONTENT (ppm)**			

Calcium (Ca)	1,900	Magnesium (Mg)	7,400
Chlorides (Cl)	470	Potassium (K)	5,200
Phosphorus (P)	14,000	Sodium (Na)	30
Iron (Fe)		80 ppm	

**VITAMIN CONTENT (μg/Gm)**			

Carotene	<.10	Pantothenic Acid	12.40
Total Tocopherols	11.00	Choline	3,270.00
Ascorbic Acid	32.00	Pyridoxine	16.40
Thiamine	3.99	Biotin	1.52
Riboflavin	4.82	Folic Acid	1.59
Niacin	83.30	Inositol	10,800

### 2.3. Growth measurement

Measurements of colony forming units were used to monitor the time course of growth in culture, as the suspended insoluble material in Pharmamedia made optical density and dry weight measurements inconvenient. In comparative experiments, cultures were passed through filter paper to collect insoluble large particles and the filtrates were passed through washed and preweighed micropore filters (0.2 μm). The cell-loaded filters were dried at 105°C and reweighed to determine the weights contributed by the cell masses. The values were calibrated by the dry weight of insoluble substances (such as iron sulfate) in fresh (noninoculated) media. However, interference by concentrated iron salts on the measurement of cell dry weight was negligible.

### 2.4. Detection and sequencing of *sfp *gene

A 675-bp fragment from the gene (*sfp*) from *B. subtilis *encoding 4'-phosphopantetheinyl transferase [57], corresponding positions 167–841 (GenBank accession no. X63158), was targeted for amplification using two oligonucleotides: P17 (5'-ATG AAG ATT TAC GGA ATT TA-3') and P18 (5'-TTA TAA AAG CTC TTC GTA CG-3'). Amplification was accomplished in a thermal cycler (Mastercycler, Eppendorf, Germany) set for denaturing at 94°C for 1 min, annealing at 46°C for 30 s, and extension at 72°C for 1 min, for a total of 25 cycles. PCR products were analyzed by 1.5% agarose gel electrophoresis. The purified PCR products were sequenced by using an ABI Prism dye terminator cycle sequencing ready reaction kit and an ABI PRISM 377 DNA sequencer (Applied Biosystems, USA). Analyses of DNA sequences were performed by using Prochromas version software (Oxford Molecular, UK).

### 2.5 Quantification and qualification analysis

#### 2.5.1 Isolation of surfactin

Crude surfactin was isolated by adding concentrated hydrochloric acid to the broth after removing the biomass by centrifugation. A precipitate was formed at pH 2 which was collected, dried, and extracted with dichloromethane. The solvent was removed under reduced pressure to give an off-white solid. Further purification was achieved by recrystallization. The dichloromethane extract was dissolved in distilled water containing sufficient NaOH to produce a pH of 8. This solution was filtered through Whatman filter paper No. 1 and titrated to pH 2 with concentrated HCl. The white solid was collected as a pellet after centrifugation. Samples were scratched out from a nonsprayed thin layer chromotography (TLC) plate extracted three times with methanol, and reduced by vacuum oven to a volume of 1 ml.

#### 2.5.2 Quantitative analysis of surfactin by HPLC

Active fractions from TLC were further purified by reversed-phase HPLC, using a Thermo Hypersil-Keystone ODS (particle size, 5 μm; column dimensions, 250 by 4.6 mm; Thermo Hypersil, USA). A sample was applied with eluent A (0.1% (vol/vol) trifluoroacetic acid and 20% (vol/vol) acetonitrile) and eluted with segmented gradients of eluent B (0.1% (vol/vol) trifluoroacetic acid and 80% (vol/vol) acetonitrile) as follows: 40% eluent B for 30 min and 40 to 100% eluent B for 10 min. Eluents A and B were made using Milli-Q HPLC grade water. The concentration of surfactin was determined from a calibration curve made by correlating the emulsification index (E 24) with known amounts of surfactin produced by *B. subtilis *ATCC 21332.

#### 2.5.3. MALDI-TOF-mass spectrometry

Fractions correlated with surfactin from TLC and Reverse phase HPLC were analyzed using MALDI-TOF-MS. 2 μL of samples were mixed on the target plate with 2 μL of matrix solution (2 mg of alpha-hydroxycinnaminic acid per ml in acetonitrile-methanol-water, 1:1:1). MALDI-TOF-MS spectra were recorded by using a 337-nm nitrogen laser for desorption and ionization. The mass spectrometer was operated in the refraction mode at an accelerating voltage of 18 kV with an ion flight path that of 0.7 m. The delay time was 375 ns. Matrix-suppression was also used, and the mass spectra were averaged over 50 to 100 individual laser shots. The laser intensity was set just above the threshold for ion production. External calibration was performed by using the [M+H]^+ ^signals of renin, adenocorticotropic hormone, insulin oxidized B, and bovine insulin (Sigma-Aldrich Co.). Surfactin isomers were anticipated to have an m/z range of 500–1500. The variance of the *m/z *of ± 0.8 Da was considered acceptable.

### 2.6. Statistical analysis

The data is presented in terms of arithmetic averages of at least three replicates, and the error bars indicate the standard deviations. The analyses were carried out using SPSS software, version 14.0 (SPSS Inc., Chicago, USA).

## 3. Results

### 3.1. Isolation and quantification of surfactin

The concentrated extracts run on silica TLC plates showed six bands under UV light, having R_f _values of 0.1, 0.15, 0.26, 0.37, 0.51 and 0.57. However, a plate bioassay showed two active fractions, those with R_f_values of 0.37 and 0.51. The spot with an R_f _value of 0.51 was ninhydrin-negative and positive to 4,4'-bis(dimehtylamino)diphenylmethane (TDM) reagent. These results indicated the absence of free amino groups and the presence of peptide bonds in the compound. A white spot formed with the same R_f _value when the plate was sprayed with water, indicating that the compound is lipophilic. The migration and chemical properties of the compound were comparable to surfactin produced by *B. subtilis *strain ATCC 21332. We observed that the environmental isolate *B. subtilis *MZ-7 produced more surfactin (170.5 mg/L) than did *B. subtilis *ATCC 21332 (109.5 mg/L) under the same conditions.

### 3.2. Detection of surfactin production by PCR

**Figure 1:**
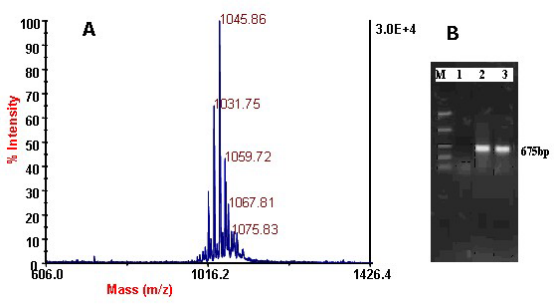
A) Surfactin cluster + Na^+ ^obtained by MALDI-TOF-MS B) Amplification of *sfp *fragments by PCR, using DNA from *B. subtilis *168, *B. subtilis *strain ATCC 21332, *B. subtilis *MZ-7 (lanes 1, 2, and 3, respectively).

*B. subtilis *subsp. *subtilis *168 was negative to specific primers for *sfp *genes. However, 675-bp fragments were amplified with genomic DNA of *B. subtilis *MZ-7 and *B. subtilis *ATCC 21332 (Figure 1B). Sequence comparisons showed 99% similarity between these fragments and *sfp *genes in the database from known surfactin producers [58].

### 3.3. Confirmation of surfactin by MALDI-TOF-MS

Running MALDI-TOF-MS in refractron mode, we observed a cluster of peaks with mass/charge (m/z) ratios between 1036 and 1058, which could be attributed to protonated surfactin isoforms (Figure 1A). The peak with a m/z ratio 1045.86 corresponds to the mass of [M+Na]^+ ^ion of surfactin with a fatty acid chain length of 14 carbon atoms [59]. Observed isomers of surfactin in different media fermentations were more or less similar [60].

### 3.4. Standardization of optimal conditions for surfactin production

**Figure 2:**
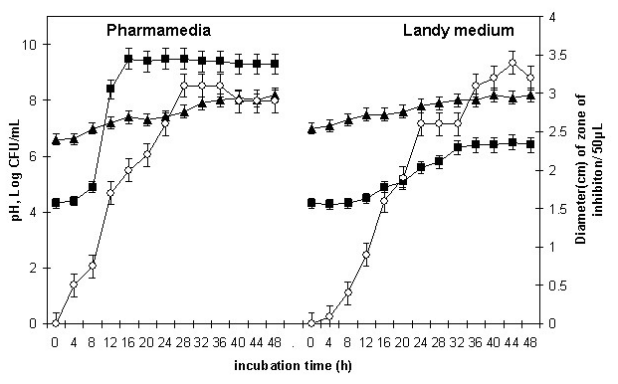
Time course of growth of *B. subtilis *MZ-7 on two growth media. Each point is the result of 3 measurements. The error bars show the standard deviation of the measurements. The following parameters were monitored: pH of the growth medium (▲), CFU (■), and ability of samples to inhibit the growth of *B. fusiformis *(○).

Figure 2 shows the relationship in Landy medium and Pharmamedia between the density of *B. subtilis *MZ-7 cultures and the pH of medium and shows the concentration of an antimicrobial compound that appeared over the time course of the incubation. In Landy medium, the concentration of the inhibitory compound was high at 24 h, when the culture was in its post-exponential phase, and reached its highest level at 44 h, i.e. at stationary phase. However, in Pharmamedia, maximum concentration was observed during the post-exponential phase (28 h), with a decrease in production during the stationary phase of the growth cycle. The optimum production time thus differs in Landy medium and Pharmamedia. The pH of culture was almost stable, ranging from 7 to 8.

### 3.5. Comparative production in different media

**Figure 3:**
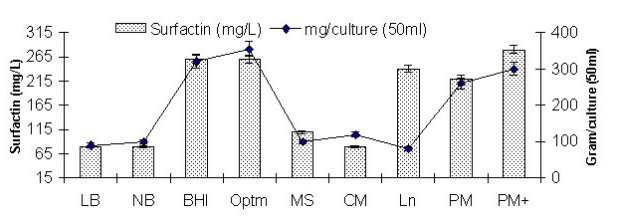
Surfactin production by *B. subtilis *MZ-7 grown on different media. Each bar represents the average of 3 measurements, with error bars indicating the standard deviation. Media used were as described in Materials and Methods: Luria bretani (LB), Nutrient broth (NB), Brain heart infusion (BHI), Optimized (Optm), Minimal salt (MS), Cooper (CM), Landy (Ln), Pharmamedia (PM), and Pharmamedia supplemented with 2 g/L sucrose and 4.0 mM Fe^+ ^(PM+).

**Figure 4:**
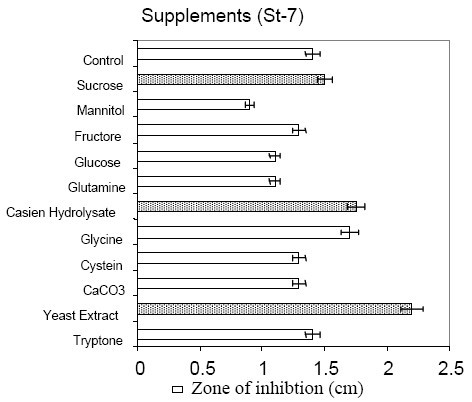
Influence of different supplements on the production of inhibitory substances (Surfactin) in comparison to non supplemented media (control).

Among the laboratory media tested (Figure 3), *B. subtilis *MZ-7 produced maximum surfactin in BHI and optimized medium (260 mg/L), followed by Landy medium (240 mg/L). Relatively low yields (220 mg/L) were observed in commercial medium (Pharmamedia) without any supplementation. Addition of various carbon and nitrogen sources were tested (Figure 4). It was observed that sucrose supplementation caused a marked enhancement in surfactin production (280 mg/L), potentially significant with regards to the goal of large scale production of surfactin on cheap media.

## 4. Discussion

Since its discovery, surfactin has proved to be an interesting biochemical tool for basic research. On account of its biodegradability and its broad range of functional properties, it is now evident that surfactin has potential applications in industrial processes, primarily those involving surfactant activities. Thus, the interest of researchers is warranted but, to date, surfactin has not been able to compete economically with its chemically synthesized counterparts because of poor strain productivity and the need for expensive substrates. Efforts must therefore be redirected to improve the production efficiency and recovery bioprocesses in order to optimize yields. Most nutritional and production studies have been done with batch cultures, usually in shake flasks, but occasionally in small-scale or large-scale fermentors [40,61-63]. A laboratory-scale cyclone column fermentor was used for continuous, phased growth with feedback control, based on the concentration of dissolved oxygen [64,65]. Saccharose and fructose have also been mentioned as efficient carbon sources, while the presence of glycerol greatly decreased surfactin production [66]. In contrast to the biosynthesis of other biosurfactants, surfactin biosynthesis was not stimulated by hexadecane [40,43].

No notable difference in the ability of different amino acids to support surfactin production has been found, yields being 600–800 mg L^-1 ^[43]. When they are used as the sole nitrogen source of a culture medium, some hydrophobic amino acids insert themselves directly into selected positions of the peptide sequence, thus amplifying the original structural microheterogeneity via the production of variants [67].

In contrast to many secondary metabolites of bacilli, the production of which is induced when the cells have exhausted one or more essential nutrients, surfactin production is induced in actively growing cells by progression to postexponential phase [11]. In most of the available literature a combination of nutritional and physicochemical factors giving the highest yields was selected for use. However, the use of effective commercial media was completely neglected until the last decades, when several scientists showed interest in using more economical, renewable sources for production [38,46]. In fact, cottonseed media have been reported to be cost-effective in the production of several antibiotics and enzymes. The performed experiments in this report were conducted in very restricted conditions in favor of reproducibility and that is to demonstrate a successful comparison not enhanced production potential. As this paper did not show economical studies, it was left for industrial economists to establish such a relation.

## Authors' contributions

Al-Ajlani carried out this research as part of his PhD work, Sheikh and Ahmad assisted in practical work and Hasnain has supervised the study.
